# Effectiveness and cost of recruiting healthy volunteers for clinical research studies using an electronic patient portal: A randomized study

**DOI:** 10.1017/cts.2018.5

**Published:** 2018-04-23

**Authors:** Mary H. Samuels, Robert Schuff, Peter Beninato, Adriel Gorsuch, James Dursch, Sarah Egan, Bridget Adams, Kate F. Hollis, Rachel Navarro, Timothy E. Burdick

**Affiliations:** 1 Oregon Clinical and Translational Research Institute, Oregon Health & Science University, Portland, OR, USA; 2 Information Technology Group, Oregon Health & Science University, Portland, OR, USA; 3 Office of the Chief Privacy Officer, Oregon Health & Science University, Portland, OR, USA; 4 University Libraries, University of Denver, Denver, CO, USA; 5 Department of Medical Informatics and Clinical Epidemiology, Oregon Health & Science University, Portland, OR, USA; 6 Departments of Community and Family Medicine and Biomedical Data Science, Geisel School of Medicine at Dartmouth, Portland, OR, USA

**Keywords:** Patient selection, healthy volunteers, electronic health records, clinical research, recruitment

## Abstract

**Introduction:**

It is not clear how to effectively recruit healthy research volunteers.

**Methods:**

We developed an electronic health record (EHR)-based algorithm to identify healthy subjects, who were randomly assigned to receive an invitation to join a research registry via the EHR’s patient portal, letters, or phone calls. A follow-up survey assessed contact preferences.

**Results:**

The EHR algorithm accurately identified 858 healthy subjects. Recruitment rates were low, but occurred more quickly via the EHR patient portal than letters or phone calls (2.7 vs. 19.3 or 10.4 d). Effort and costs per enrolled subject were lower for the EHR patient portal (3.0 vs. 17.3 or 13.6 h, $113 vs. $559 or $435). Most healthy subjects indicated a preference for contact via electronic methods.

**Conclusions:**

Healthy subjects can be accurately identified from EHR data, and it is faster and more cost-effective to recruit healthy research volunteers using an EHR patient portal.

## Introduction

Inadequate recruitment into clinical research studies is a major cause of failure to meet study goals, and leads to a considerable drain on institutional resources. It delays medical progress, limits data for publications and grants, and diverts resources from more productive pursuits. In a recent analysis of 374 studies at Oregon Health & Science University (OHSU), 31% of clinical research studies requiring full Institutional Review Board (IRB) review enrolled 0–1 subject before being terminated. Over $1 million per year was wasted on these studies [1]. The underlying causes of under-enrollment are multi-factorial, but include inadequate time, experience, and resources to recruit subjects effectively [2, 3].

A recent Institute of Medicine report on the National Institute of Health’s Clinical and Translational Science Award (CTSA) program emphasized the need for novel informatics tools to help accelerate the progress of clinical research, including for recruitment [4]. In this regard, progress has been made in leveraging electronic health record (EHR) systems to recruit research participants. These efforts involve 2 steps: cohort identification, querying an EHR using a predetermined screening algorithm to generate a list of potentially eligible subjects; and outreach to identified subjects to ascertain whether they are interested and in fact eligible for the study, which can be accomplished manually or using automated systems tied to the EHR [3]. Published reports have described EHR-based recruitment strategies in different clinical domains, particularly oncology [3]. However, few studies have directly compared traditional and EHR-based research recruitment strategies for effectiveness, efficiency, and quality of enrolled participants [3].

Many clinical research studies require the participation of patients with specific diseases or phenotypes, but others are targeted to healthy populations, either as primary research subjects, or as control subjects for comparison with patients. There are further hurdles to recruiting healthy research subjects, as they have less contact with the medical profession, and may be less likely to volunteer if they have no vested interest in research outcomes.

There is no published information on the ability of EHRs to facilitate the recruitment of healthy research volunteers. There are obvious reasons why an EHR may not be optimal for this function, since most patients seen in clinical settings have chronic illnesses and are on medications. However, some patients are seen for preventive care and/or acute, self-limiting illnesses. An EHR-based algorithm that accurately identifies such subjects would need to be based on exclusion, rather than inclusion, criteria, which presents technical challenges. Even if these subjects are accurately identified using an algorithm, it is not clear that they would consider it acceptable to be contacted for research studies, and what would be the best method to reach out to them.

To facilitate investigator access to potential research volunteers and biospecimens, the OHSU Oregon Clinical and Translational Research Institute (OCTRI, which is OHSU’s CTSA site) maintains an IRB-approved Research Volunteer Registry (RVR). The RVR includes a database of preconsented participants willing to be contacted at later dates to provide biospecimens or volunteer for research procedures. We decided to utilize the OHSU EHR and OCTRI RVR to test the following hypotheses:

1. Substantial numbers of healthy subjects can be accurately identified by querying the EHR. We designed an algorithm using diagnosis codes and medication lists to address this question, with manual review of a subset of records to confirm accuracy.
2. It is more effective, faster, and less expensive to recruit healthy subjects into the RVR by direct contact through the EHR secure portal, compared with traditional recruitment methods of letters or phone calls. We designed a randomized study of subjects identified as healthy in our EHR algorithm to address this question.
3. Healthy subjects find it acceptable to receive research recruitment messages at specified intervals via direct contact through the EHR secure portal. We designed a follow-up survey of subjects identified as healthy in our EHR algorithm to address this question.

## Methods

### Brief Description of the RVR

#### Inclusion and Exclusion Criteria

Inclusion criteria for the OCTRI RVR are age 18–89 years; have phone or email and are willing to be contacted periodically to update their information; and are able to provide informed consent. Exclusion criteria are serious medical conditions or medications, including cardiac or pulmonary disease, diabetes mellitus, cancer, active infectious disease, and active inflammatory diseases. Other exclusions are based on the medical judgment of the RVR principle investigator (M.H.S.).

#### Recruitment into the RVR

Various IRB-approved recruitment methods are used for the RVR, including health fairs, direct advertisement, and referral from other OHSU IRB-approved studies. The RVR includes an IRB-approved consent form, completed in person or online via a brief, secure REDCap survey. REDCap is a widely deployed secure web-based data collection and management application for building and managing online surveys and databases, especially for research activities (https://www.project-redcap.org/) [5]. Consented participants are provided with a link to a REDCap enrollment survey, or an option to complete the survey on paper or via phone. The survey includes demographic information, medical diagnoses, medications, and contact preferences. The information is confirmed via a follow-up phone call with an RVR study coordinator. A subset of enrolled RVR volunteers is invited to an in-person visit to donate biological samples for storage. Enrolled volunteers are re-contacted annually to indicate their continued willingness to participate and to update their information.

#### Release of RVR Data

When investigators request volunteer demographic and medical information or precollected biospecimens, the repository staff verifies that all IRB rules are followed, and removes private health information if the request is for de-identified data. Data or biological samples are then released to the investigator for a small fee to cover RVR maintenance costs.

### Development of the EHR Algorithm Using Diagnosis Codes and Medications

The EHR algorithm was developed and implemented in Epic, OHSU’s EHR (Epic Systems, Verona, WI). OHSU has a single instance of the Epic EHR integrating ambulatory, inpatient, and other clinical modules and with billing and administrative modules. OHSU deployed Epic ambulatory in 2005 while inpatient and Emergency Department modules were deployed in 2008, providing a rich data set of over a decade’s duration.

Three of the investigators (M.H.S., T.E.B., P.B.) developed the algorithm through an exclusionary approach, seeking to exclude patients whose records contained diagnosis codes or medications indicating chronic or debilitating conditions. Diagnosis codes examined were those associated with Admit, Medical History, Encounter, or Primary diagnoses. Since the study occurred during OHSU’s transition from ICD-9 to ICD-10 codes, either ICD-9 or ICD-10 codes were considered part of the inclusion/exclusion diagnoses. Medications examined were medication orders and medications listed on the patients’ current medications list.

We first defined a set of diagnosis codes acceptable for inclusion (primarily minor and self-limited conditions) and then determined the complement of this set, producing a set of exclusionary criteria. Medication inclusion criteria were similarly developed based on therapeutic class and availability as an over-the-counter medication. Acceptable medications included allergy medications, non-steroidal anti-inflammatory agents, oral contraceptives, antibiotics for acute infections, and acetaminophen. The complement of this set of medications was then derived and applied to develop the set of patients to be excluded. This set of exclusionary criteria was then applied to all otherwise included patients and the resulting patient set was eliminated resulting in a population of patients deemed “healthy.” A total of 659 parent ICD-10 codes were acceptable for inclusion in the algorithm; the ICD-10 codes were also matched to ICD-9 codes, such that both ICD-9 and ICD-10 exclusionary codes were used.

Patients were included if they were 21–89 years of age, and had at least one in-person encounter within the prior 5 years. We restricted patients to those living in Oregon and Washington, since our goal was to recruit volunteers who could travel to OHSU for research studies. Finally, we excluded patients from 1 major OHSU clinical department due to reluctance on the part of its faculty to have their patients receive MyChart messages regarding research studies.

### Manual Review of EHR Records to Confirm Accuracy of the Algorithm

One of the investigators (M.H.S.) manually reviewed the complete EHR charts from a random sample of 48 subjects identified by the algorithm (5.5% of the total). Charts were reviewed using the same inclusion and exclusion criteria as the algorithm, with the addition of review of free text from provider notes.

### Comparison of Methods to Recruit Subjects from the EHR Algorithm into the RVR

The EHR algorithm-generated list of 858 healthy subjects was divided in a randomized fashion into 3 groups. Due to resource constraints, subjects were randomized in a 4:2:1 ratio, with fewer subjects in the more time-intensive groups. Groups were matched for age (within 5 years), gender, and race/ethnicity. The text of the recruitment documents for the 3 groups was as similar as possible, to allow for direct comparison of success rates. All 3 recruitment methods are actively in use at OHSU, and the OHSU IRB approved the study and all contact documents.

- In total, 482 subjects were recruited via an IRB-approved MyChart message. MyChart is Epic’s secure, HIPAA-compliant email portal used for communications between patients and OHSU providers. OHSU patients sign up for MyChart accounts on a voluntary basis. OHSU has over 130,000 patients with active MyChart accounts, with over 2000 new users each month. OHSU’s Epic system enables specific MyChart messages to be sent directly to potential research volunteers who have active MyChart accounts. The MyChart message was formatted as an invitation to join the RVR, with a link to the IRB-approved RedCap electronic RVR survey. There was an opt-out link for subjects who did not wish to be contacted for future studies via MyChart. Only 1 MyChart message was sent to this group.
- In total, 237 subjects were recruited via a standard IRB-approved letter, containing information about the RVR and a request to log into the RedCAP survey or call the RVR study coordinator. Per standard RVR procedures for letter-based recruitment, 1 follow-up phone call was made to subjects who did not respond to the letter within 4 weeks.
- In total, 139 subjects were contacted via a phone call, using an IRB-approved phone script that contained information about the RVR and a request to return the phone call if subjects were interested but not available for the initial phone call. Per standard RVR procedures for phone-based recruitment, 1 follow-up phone call was made to subjects who did not respond to the initial phone call within 4 weeks.

For each of the 3 recruitment methods, we tracked number of subjects enrolled into the RVR, speed of recruitment, and the amount of time spent per enrollment. The latter included the number of hours of work required to generate the letters, phone calls, and MyChart messages, as well as follow-up activities to verify eligibility and enter data into the REDCap database. Costs were calculated based on our standard rate of $32 per hour for study coordinator time, and $0.37 for postage per letter sent. An additional one-time charge of $395 was applied only to the MyChart recruitment arm, which reflected 5 hours of time (at $79/h) by the OHSU Epic Research Team to work with the study coordinators to craft and send out the 482 MyChart messages. Not included was the initial informatics effort to generate and validate the EHR algorithm, or time spent on IRB-related activities for the project, since these were equally applied across all 3 methods.

### Follow-Up Survey

To ascertain subjects’ opinions of the 3 recruitment methods, we sent a follow-up IRB-approved REDCap survey to all 858 subjects 1 month after completion of recruitment. Surveys were sent via the same method of contact as the initial message. The survey asked subjects to rate their experience of being recruited and their willingness to be contacted for research studies via different methods at specified intervals.

### Statistical Analysis

Continuous variables were compared among the 3 groups by analysis of variance with post-hoc Tukey honest significance difference (HSD) test. Categorical variables were compared by χ^2^ or Person Time Rate test, with post-hoc Bonferroni adjustments.

## Results

### Description of the Epic Algorithm

We initially included 389,768 patients aged 21–89 years who had an in-person encounter (19 encounter types) within the prior 5 years. Of these 389,768 patients, 949 were identified as healthy and had active MyChart accounts (defined as having used their MyChart accounts within the past 2 y). In total, 356,095 patients were excluded due to diagnosis and 348,669 excluded due to medications (most of whom also had excluded diagnoses). One department did not want its patients contacted through MyChart resulting in a further exclusion of 91 patients with their primary care provider credentialed in that department. This resulted in the identification of 858 potential healthy subjects.

Ten of the 482 subjects contacted via MyChart opted out of future contact for research studies (2%).

Manual review of medical records from a random selection of 48 RVR-eligible subjects identified by the EHR algorithm identified one subject who had epilepsy. This information was only recorded in the clinical note as free text, and was not present in the patient’s diagnoses, problem list, or past medical history.

### Randomized Study Results (Table 1)

By design, mean ages (37.8–38.4 y) percentages of women (58%), ethnicity (86%–91% non-Hispanic/Latino), and race (75%–78% White) were similar among the 3 groups (Table 1).

**Table 1:** Research Volunteer Registry (RVR) enrollment rates and hours of effort by method of initial contact

	MyChart	Letters	Phone calls
No. of subjects contacted	482	237	139
Age (y)	37.8±0.8	38.1±0.8	38.4±1.0
Gender (% female)	58	58	58
Ethnicity (% not Hispanic/Latino)	90	91	86
Race (% White)	78	78	75
No. of contact attempts	482	464	256*
No. of subjects enrolled in RVR (% of subjects contacted)	23 (4.8%)	14 (5.9%)	12 (8.6%)
No. of subjects enrolled in RVR (% of contact attempts)	23 (4.8%)	14 (3%)	12 (5%)
Speed of enrollment (d)	2.7±1.1	19.3±3.6**	10.4±3.1**
Effort per enrolled subject (h)	3.2	17.3***	13.6***
Cost per enrolled subject ($)	113	559***	435***

Values are means±SEM for continuous variables. Speed of enrollment was defined as no. of days from initial contact to REDCap enrollment. Enrollment rates were not different among the 3 groups by χ^2^ (*p*=0.22 by number of subjects contacted and *p*=0.34 by number of contact attempts). *p<0.00001 compared to MyChart and letters by χ^2^ with post-hoc Bonferroni adjustment. **p<0.01 compared to MyChart by analysis of variance with post-hoc Tukey HSD test. ***p<0.001 compared to MyChart by Person Time Rate with post-hoc Bonferroni adjustment.

Overall recruitment rates were low for all 3 methods, and were not different among the methods (4.8% of subjects contacted by MyChart, 5.9% by letter, and 8.6% by phone call, *p*=0.22). Recruitment rates were also similar by number of contact attempts (3%–5% for all methods, *p*=0.34). Enrollment occurred more quickly via MyChart than the other 2 methods (mean of 2.7 d for MyChart, 19.3 d for letters, and 10.4 d for phone calls, *p*<0.01). In total, 17 subjects contacted via MyChart (74%) enrolled within 1 day, compared with none of the subjects contacted via letter and 4 of the subjects contacted by phone (33%). The longest time lag before a subject enrolled was 19 days after receiving the MyChart message, 57 days after receiving a letter with a follow-up phone call, and 37 days after receiving initial and follow-up phone calls. Hours of effort per enrolled subject were 3.0 by MyChart, 17.3 by letter, and 13.6 by phone call (*p*<0.0001). Costs per enrolled subject were $113 for MyChart, $559 for letter, and $435 for phone call (*p*<0.0001).

### Follow-Up Survey Results (Table 2, Fig. 1)

All 482 subjects who had received initial MyChart messages were contacted with a request to complete the follow-up survey. Two hundred twenty-three of 237 subjects who received initial letters were contacted via letter for the follow-up survey; the remaining subjects did not have current addresses. One hundred eight of 139 subjects who received initial phone calls were contacted via phone calls for the follow-up survey; the remaining subjects did not have working phone numbers.

Response rates to the survey were low, with only 20 MyChart subjects (4%) and 42 letter, or phone call subjects (12%) completing the survey by the same method as their original contact method (Table 2). Due to these low numbers, results for letter and phone call subjects were combined for analysis. In all, 18 MyChart subjects (90%) indicated that it was acceptable to be contacted via MyChart for research studies, compared with 24 (57%) of letter or phone call subjects (*p*=0.01). None of the MyChart subjects and only 7% of the other subjects indicated it was not acceptable to be contacted via MyChart for research studies (*p*=NS). One MyChart subject and 8 other subjects indicated that they did not use MyChart, despite the fact that they all had active OHSU MyChart accounts.

**Table 2:** Follow-up survey responses to the question “Was it (or would it be) acceptable to you to be contacted using a MyChart message for a research study?”

	Original contact via MyChart	Original contact via letters or phone calls
No. of responses to follow-up survey (% of original subjects contacted)	20 (4%)	42 (11%)
No. of responders who indicated it was acceptable to be contacted via MyChart for research studies (% of responders)	18 (90%)	24* (57%)
No. of responders who indicated it was not acceptable to be contacted via MyChart for research studies (% of responders)	0 (0%)	7 (17%)
No. of responders who were not sure it was acceptable to be contacted via MyChart for research studies (% of responders)	1 (5%)	3 (7%)
No. of responders who indicated they did not use MyChart (% of responders)	1 (5%)	8 (19%)

*p<0.01 compared to MyChart group by χ^2^.

**Figure 1:**
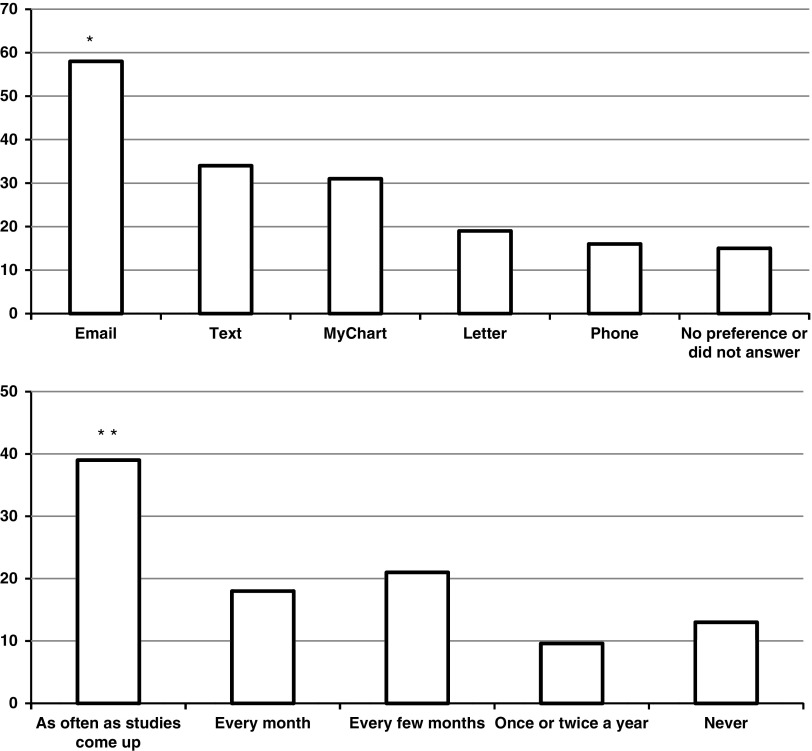
Top: percent of subjects who responded to the follow-up survey who ranked the listed method as their first or second choice to be contacted about research studies. *p<0.001 compared to letter, phone, or no preference by χ^2^ with post-hoc Bonferroni adjustment. Bottom: percent of subjects indicating what frequency of contact for future research studies would be acceptable in the follow-up survey. **p<0.01 compared to “once or twice a year” or “never” by χ^2^ with post-hoc Bonferroni adjustment.

Subjects were asked to rank their preferences for being contacted for future research studies (Fig. 1, top). They were given 5 choices (email, MyChart message, text message, letter, or phone call). The numbers of responders who ranked a method 1 or 2 were 36 (58%) for direct email, 21 (34%) for text messages, 19 (31%) for MyChart messages, 12 (19%) for letters, and 10 (16%) for phone calls. The number of responders indicating a preference for email was significantly greater than for letters or phone calls (*p*<0.001).

Finally, subjects were asked how often it would be permissible to contact them about research studies in the future (Fig. 1, bottom). In all, 24 (39%) indicated it would be acceptable to be contacted as often as studies came up (*p*<0.01 compared with “once or twice a year” or “never”). Fewer numbers of subjects indicated it would be acceptable to be contacted at less frequent intervals, and 8 subjects (13%) indicated that they did not want to be contacted.

## Discussion

Electronic patient data management systems, including EHRs, are increasingly being utilized to identify potential research volunteers with specific diseases [6–14]. However, many research studies require healthy volunteers, who can be challenging to recruit. To our knowledge, our study represents the first attempt to systematically identify healthy subjects from an EHR using an automated algorithm. We found that healthy subjects can be accurately identified using an algorithm that excludes all but a small number of ICD-10 diagnostic codes and medications. Manual review of a subset of identified records found a 2% false-positive rate (inclusion of a subject with an excluded diagnosis), which is well within an acceptable pre-screening rate. That subject was identified through review of unstructured (free text) data, which is not currently included in most EHR-based screening algorithms [3]. Due to resource constraints, it was not feasible to ascertain the false-negative rate (missed healthy subjects), as this would have involved reviewing large numbers of patient records not tagged by the algorithm. However, the false-negative rate is less relevant if sufficient numbers of healthy subjects are correctly included in the algorithm-generated data. We found 858 subjects, which is an under-count of actual healthy subjects in our EHR, since one major OHSU clinical department declined to allow us to contact their patients. They had concerns about patient privacy and extra workload for their care providers, despite the fact that only 2% of contacted subjects opted out of future contacts, and our follow-up survey indicated broad acceptance from subjects.

Identifying potential research volunteers via an EHR algorithm is only the first step in recruitment. Potential volunteers must be contacted to see if they are interested in participating and are actually eligible for the study. In this regard, our study is the first to rigorously compare electronic and traditional recruitment methods for any group of research subjects via a randomized, parallel approach. Our overall recruitment effectiveness was low for all 3 methods, likely reflecting the fact that healthy subjects have less interest in participating in research studies compared with patients. On the other hand, direct contact via MyChart, our secure email-based patient portal, was much faster and less costly than traditional methods of contact via letters or phone calls. This may also be true for recruiting patients with specific diseases, although that may vary depending on the population of available patients and their relationship with the health care system. Previous studies have reported highly variable costs for recruitment using traditional (non-electronic) methods, but have not directly compared methods using matched groups, as in our study [15–19].

In terms of overall costs, we did not include the initial costs of creating the inclusion/exclusion criteria and identifying patients from the RDW, as that work covered all 3 recruitment methods equally. The 2 physician investigators (M.H.S., T.E.B.) created the ICD-9/ICD-10 list over the course of ~3 hours. The IT specialist (P.B.) spent ~20 hours creating the Epic algorithm and refining the list of subjects from Epic. We did include the extra time required for IT consultation (5 h) to facilitate the MyChart messages. Also not included are general ongoing equipment and maintenance costs for IT systems associated with EHRs; without these systems, MyChart recruitment would obviously be impossible, but these general costs are difficult to apply to a cost analysis of a specific project such as this one. Such costs would incrementally decrease the cost difference between MyChart and other recruitment methods, but we do not believe they would obviate the cost advantage of MyChart. In addition, most large medical centers either have or are in the process of establishing EHRs, and marginal costs for this type of activity would be expected to be modest in that context.

We found 9 published studies that compared recruitment outcomes between manual and computerized support systems [6–14]. They each focused on specific inpatient or outpatient populations with acute or chronic illnesses; none included healthy control subjects. Most utilized historical control time periods, and none were randomized by recruitment method. All of these studies reported enhanced recruitment rates and/or decreased time and costs of recruitment using computerized support systems. Our results are not directly comparable to these studies, given the different patient populations, study designs, and electronic systems, but our results are similar in terms of faster and less expensive recruitment via an EHR.

In all of these previous studies, potential research subjects were identified by commercial or in-house computerized support systems, but actual recruitment occurred using patient lists, emails, pages, or alerts sent to physicians or coordinators, who then performed manual record review before approaching potential subjects. As shown by our results, direct contact of potential subjects via a secure EHR patient portal circumvents this added workload and provides additional cost and effort savings, without sacrificing recruitment rates or subject satisfaction. This also avoids having to engage busy care providers who may ignore or resent interruptions in workflow, or may develop “alert fatigue” [2]. However, successful implementation at our institution required extensive efforts to educate providers and staff before the system was broadly acceptable. We found that the data from our randomized study and follow-up survey were helpful in reassuring providers and institutional leaders that this method enhances research progress while being acceptable to most patients, as shown by our follow-up survey.

We could find only one previous study that described strategies for recruiting healthy volunteers [20], which compared 2 methods to find volunteers to test a telephone-based education program in emergency cardiac massage: a previously developed healthy volunteer database Versus posters and media advertisements aimed at the general public. Ten percent of the subjects they called from their database enrolled in the study, while they had little success with posters and media advertisements. We created our RVR to provide a similar function for multiple studies needing healthy volunteers, and our data suggest that EHR-based recruitment strategies to develop healthy volunteer databases is a viable and cost-effective process.

Harris *et al*. previously described their experience with developing a Web-based recruitment model to allow potential research volunteers to register for current and future studies at Vanderbilt University Medical Center [21]. Subjects utilized a secure internet connection to self-enter personal information, which investigators could then access to contact potential volunteers. Although this registry was not focused on healthy subjects, they did include the question “Are you a “normal volunteer?”. They found that 75% of subjects answered “yes” to this question, but most also listed medical conditions and medications that excluded them from a healthy volunteer list. We had a similar experience with our RVR, as manual review of volunteers’ self-entered data revealed many such examples. Therefore, we do not believe that we can avoid a manual secondary review of data entered by volunteers, which ensures that investigators actually receive control data and biological samples as requested. This adds time and expense to establishing and maintaining an RVR, but assures its fidelity.

Our study had a number of strengths: we successfully developed and tested an EHR-based algorithm to identify healthy subjects from an academic medical center patient population. We used this algorithm-generated data to measure efficacy and costs utilizing 3 recruitment methods in a randomized fashion, including direct contact via our EHR’s secure portal. Finally, we employed a follow-up survey to measure subjects’ acceptance of the different recruitment methods. However, our study also had some limitations: our results were subject to limitations inherent in EHR data, which inevitably contain errors and omissions [3]. Despite that, we found a very low false-positive rate. We could not query all OHSU patients due to one clinical department’s reluctance to allow us to directly contact their patients, which limited our sample size and may have reduced our ability to find differences among the 3 contact methods. Not all subjects had the same number of contact attempts, which depended on the method and our standard workflow. However, we reached diminishing returns for repeated contact attempts, which increased costs without increasing enrollment rates. Response rates to our follow-up survey were very low, limiting interpretation of those results. OHSU’s patient population demographic is largely non-Hispanic White, and we cannot generalize our results to minority populations, who may be less likely to volunteer for research studies in general. The mean age of healthy subjects in our study was 38 years, likely reflecting younger patients’ greater comfort with electronic communication tools, and it is not clear that recruiting older subjects via an electronic patient portal would be effective.

In conclusion, we report for the first time a successful EHR-based algorithm to identify healthy subjects in sufficient numbers to provide a platform for recruitment into research studies. We utilized the resulting list of potential volunteers to rigorously test 3 approved recruitment strategies for a healthy volunteer repository. We found that recruitment rates were low for all methods, but direct contact with subjects via a secure electronic patient portal was much faster and less expensive than traditional methods of letters or phone calls. We were particularly impressed with the speed with which some subjects enrolled via the MyChart message, sometimes within minutes to hours of receiving the message. This could be especially powerful for studies that require rapid or competitive enrollment. Direct contact was also acceptable to most patients at frequent intervals. In the hopes of assisting other institutions meet the needs of clinical investigators, we have made this algorithm broadly available at no charge to Epic users: diagnosis codes list: https://userweb.epic.com/Thread/61473; clarity SQL: https://userweb.epic.com/Thread/61634
